# Simplified and optimized multispectral imaging for 5-ALA-based fluorescence diagnosis of malignant lesions

**DOI:** 10.1038/srep25530

**Published:** 2016-05-05

**Authors:** Takeo Minamikawa, Hisataka Matsuo, Yoshiyuki Kato, Yoshinori Harada, Eigo Otsuji, Akio Yanagisawa, Hideo Tanaka, Tetsuro Takamatsu

**Affiliations:** 1 Department of Pathology and Cell Regulation, Graduate School of Medical Science, Kyoto Prefectural University of Medicine, 465 Kajii-cho Kawaramachi-Hirokoji, Kamigyo-ku, Kyoto 602-8566, Japan; 2 Division of Digestive Surgery, Department of Surgery, Graduate School of Medical Science, Kyoto Prefectural University of Medicine, 465 Kajii-cho Kawaramachi-Hirokoji, Kamigyo-ku, Kyoto 602-8566, Japan; 3 Ushio Inc., 6409 Moto-Ishikawa-cho, Aoba-ku, Yokohama, Kanagawa 225-0004, Japan; 4 Department of Surgical Pathology, Graduate School of Medical Science, Kyoto Prefectural University of Medicine, 465 Kajii-cho Kawaramachi-Hirokoji, Kamigyo-ku, Kyoto 602-8566, Japan; 5 Department of Medical Photonics, Kyoto Prefectural University of Medicine, 465 Kajii-cho Kawaramachi-Hirokoji, Kamigyo-ku, Kyoto 602-8566, Japan

## Abstract

5-aminolevulinic acid (5-ALA)-based fluorescence diagnosis is now clinically applied for accurate and ultrarapid diagnosis of malignant lesions such as lymph node metastasis during surgery. 5-ALA-based diagnosis evaluates fluorescence intensity of a fluorescent metabolite of 5-ALA, protoporphyrin IX (PPIX); however, the fluorescence of PPIX is often affected by autofluorescence of tissue chromophores, such as collagen and flavins. In this study, we demonstrated PPIX fluorescence estimation with autofluorescence elimination for 5-ALA-based fluorescence diagnosis of malignant lesions by simplified and optimized multispectral imaging. We computationally optimized observation wavelength regions for the estimation of PPIX fluorescence in terms of minimizing prediction error of PPIX fluorescence intensity in the presence of typical chromophores, collagen and flavins. By using the fluorescence intensities of the optimized wavelength regions, we verified quantitative detection of PPIX fluorescence by using chemical mixtures of PPIX, flavins, and collagen. Furthermore, we demonstrated detection capability by using metastatic and non-metastatic lymph nodes of colorectal cancer patients. These results suggest the potential and usefulness of the background-free estimation method of PPIX fluorescence for 5-ALA-based fluorescence diagnosis of malignant lesions, and we expect this method to be beneficial for intraoperative and rapid cancer diagnosis.

Fluorescence detection based on 5-aminolevulinic acid (5-ALA)-induced protoporphyrin IX (PPIX) fluorescence has recently emerged as a promising method for ultrarapid intraoperative detection of malignant lesions^1^,^2^,^3^,^4^,^5^,^6^. 5-ALA-based fluorescence detection of malignant lesions is now clinically applied in brain surgery^4^,^7^, urological surgery^8^,^9^,^10^, and gastrointestinal surgery^11^,^12^. This fluorescence method is based on selective accumulation of PPIX, which is a fluorescent metabolite of 5-ALA, due to altered activity of enzymes of cancer cells, the increase in porphobilinogen deaminase activity and the reduction in ferrochelatase activity^13^,^14^,^15^,^16^. Although several researchers reported the efficacy of the 5-ALA-based fluorescence detection method in clinical application, detection errors often appear due to strong background of autofluorescence of chromophores. To improve the detection accuracy of the 5-ALA-based fluorescence detection in the presence of strong autofluorescence, several spectral analytical methods were proposed^17^,^18^,^19^,^20^,^21^,^22^. Although the conventional methods highlighted PPIX fluorescence against background noise of autofluorescence, these either take a long time for acquiring images or autofluorescence elimination is not sufficient.

In this study, to overcome these disadvantages of the conventional methods, we propose a simplified multispectral imaging method for quantitative and sensitive 5-ALA-based fluorescence detection of malignant lesions, especially for lymph node metastasis. By optimizing observed wavelength for the fluorescence detection by computational simulation, we attained robust and quantitative estimation of PPIX fluorescence intensity against unwanted autofluorescence with three spectral images. We also demonstrated detection capacity of our method by using lymph node metastases of colorectal cancer patients.

## Results

### Optimization of observation filters for quantitative detection of PPIX fluorescence by multispectral imaging

To optimize observation wavelength for PPIX fluorescence detection, we sought the wavelength with minimum estimation error of PPIX fluorescence intensity under the condition of varied intensity ratio of PPIX fluorescence and adjacent autofluorescence. Furthermore, robustness against observation noise such as shot noise and thermal noise of the detector is important for reliable detection of PPIX fluorescence. We focused on two sources of predominant autofluorescence of lymph node metastasis, derived from flavin adenine dinucleotide (FAD) and collagen, and sought to optimize the observation wavelength in the presence of random noise.

**Figure 1:**
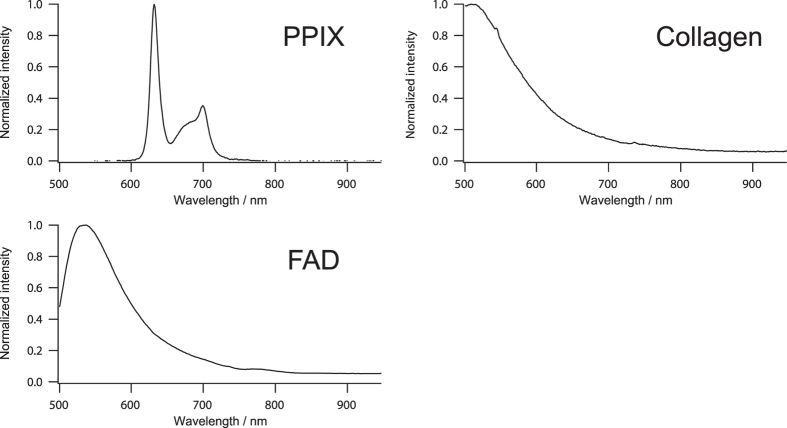
Representative fluorescence spectra of PPIX, FAD, and collagen.

On the computational optimization of observation wavelength, we used representative fluorescence spectra of PPIX, FAD, and collagen as shown in Fig. 1. Since the fluorescence spectrum of the pure chemical from of PPIX dissolved in DMSO was almost identical with that of the 5-ALA-induced PPIX that was demonstrated with a mouse rectal cancer model after 5-ALA treatment in our previous study^23^, we employed the fluorescence spectrum of the pure chemical from of PPIX dissolved in DMSO for the computational optimization. The normalized representative fluorescence spectra with the peak intensities of 0, 50, and 100 of each chemical species were used as base spectra. Linear combinations of these base spectra were constructed as a data set (total of 27 patterns). One hundred patterns of randomly generated Gaussian noise with standard deviation of intensity of 5 were additionally applied to each linearly combined fluorescence spectrum, and then a total of 2,700 spectra were used for the optimization model.

To optimize the observed wavelength, we firstly constructed a regression model:


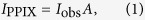


where *I*_PPIX_, *I*_obs_, and *A* represent a matrix of true PPIX fluorescence (n × 1), a matrix of observed intensity data set (n × m + 1), and regression coefficient (m + 1 × 1), respectively. The number of regression data with various values of *I*_PPIX_ and the number of observation wavelength regions for the prediction of PPIX fluorescence are respectively defined as n and m. We chose m wavelengths as the observed intensity data set, and so the regression coefficient has (m + 1) components including a constant term. The regression coefficient *A* was calculated with Moore-Penrose pseudo inverse *I*^+^:


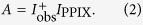


Then we calculated prediction intensity of PPIX fluorescence *I*_pred_ with the calculated regression coefficients and equation (1). The optimization model was finally illustrated as follows:


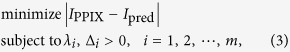


where *λ*_*i*_, and Δ_*i*_ are center observation wavelengths and these full width and half maximum (FWHM), respectively. In mathematical consideration, three or more linearly independent observations are at least required for the estimation of PPIX fluorescence intensity in the complex mixture of three components (PPIX, FAD, and collagen) with a unique solution. In contrast, total observation time will be prolonged by increasing the number of observation wavelength regions. In this study, we thus determined the number of observation wavelengths as three wavelengths for simplified but reliable estimation of PPIX fluorescence.

**Figure 2:**
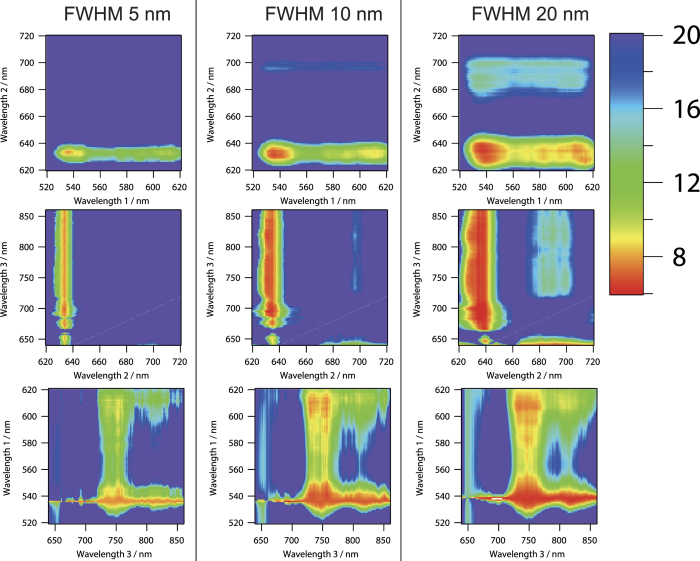
Computational simulation of standard estimation error of PPIX fluorescence intensity by varying the observation wavelengths with the fixed FWHMs of 5 nm, 10 nm, and 20 nm. Optimum center wavelengths were 536, 634, and 742 nm in the FWHM of 5 nm, 536, 634, and 746 nm in the FWHM of 10 nm, and 538, 635, and 745 nm in the FWHM of 20 nm. Another wavelength axis on each figure was fixed at the optimum wavelength. Minimum values of the standard estimation error of each FWHM were 7.82, 6.98, and 6.40 in the FWHM of 5 nm, 10 nm, and 20 nm, respectively. Color bar represents standard estimation error.

Computational simulation results of standard estimation error of PPIX fluorescence intensity by varying the observation wavelengths with fixed FWHM are shown in Fig. 2. The standard estimation error was defined as root mean square of the difference of actual and predicted PPIX fluorescence intensities in the case of the spectra data set described above (data set for total of 2,700 spectra). Owing to the PPIX fluorescence distribution from 620 nm to 720 nm, we sought the following wavelength regions for the optimization: from 520 to 630 nm with 1 nm step as observed wavelength 1, from 620 to 720 nm with 1 nm step as observed wavelength 2, and from 640 to 860 nm with 1 nm step as observed wavelength 3. The distributions of standard estimation error of PPIX fluorescence were similar among the results when we set the FWHMs at 5, 10, and 20 nm. In wavelength 2, small value of the standard estimation error was obtained around 635 and 700 nm. These two wavelength regions coincided with the peak intensities of PPIX fluorescence. The standard estimation error around 635 nm was smaller than that around 700 nm. In contrast, small values of the standard estimation errors of wavelength 1 and wavelength 3 were broadly obtained from around 530 to 610 nm in wavelength 1 and from around 680 to 850 nm in wavelength 3. In these regions, both of the spectral shapes of FAD and collagen were monotonically decreasing. These results may suggest that one possible observation wavelength should be high fluorescence intensity region of PPIX, and the wavelength region with monotonous change of spectral shape of autofluorescence is better for the other two observation wavelengths. In these wavelength regions, the optimum center wavelengths were 536, 634, and 742 nm in the FWHM of 5 nm, 536, 634, and 746 nm in the FWHM of 10 nm, and 538, 635, and 745 nm in the FWHM of 20 nm. Minimum values of the standard estimation error of each FWHM were 7.82, 6.98, and 6.40 in the FWHM of 5 nm, 10 nm, and 20 nm, respectively.

**Figure 3:**
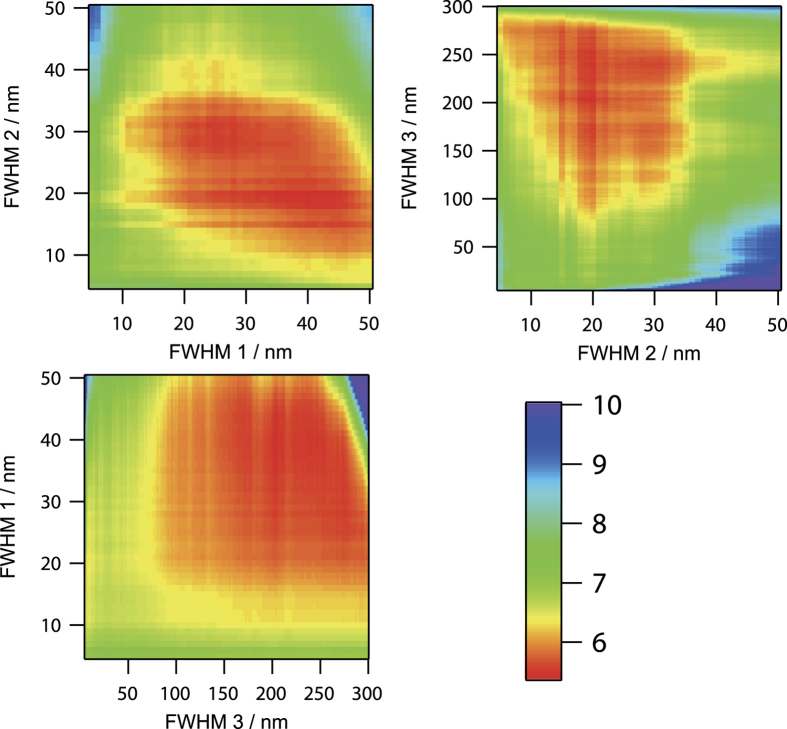
Computational simulation of standard estimation error of PPIX fluorescence intensity by varying the FWHMs of the observation wavelengths. The observation center wavelengths were set at the optimum wavelength of 536, 634, and 745 nm. The minimum standard estimation error of PPIX fluorescence was 5.44 at the observation wavelengths of 536 nm with FWHM of 40 nm, 634 nm with FWHM of 20 nm, and 745 nm with FWHM of 204 nm. FWHM 1, FWHM 2, and FWHM 3 indicate the FWHM of observation wavelength 1, wavelength 2, and wavelength 3, respectively. Color bar represents standard estimation error.

Since the optimum center wavelengths of each fixed FWHM shown in Fig. 2 were distributed near 537, 635, and 744 nm, we performed further optimization by varying the FWHM of observation wavelength with varying of the center wavelength near 537, 635, and 744 nm. For the optimization of FWHM of each observed wavelength, we varied from 4 to 50 nm for wavelength 1 (around 537 nm) and wavelength 2 (around 635 nm), and from 4 to 300 nm with 1 nm step for wavelength 3 (around 744 nm). Computational simulation results of standard estimation error of PPIX fluorescence intensity by varying the FWHM of observation wavelengths with fixed center wavelength are shown in Fig. 3. In the FWHM of the observation wavelength 2, small value of standard estimation error was obtained at less than 40 nm of FWHM. This wavelength region almost coincided with the width of the first fluorescence peak of PPIX at around 635 nm. This result may indicate that the contribution of the noise of the unwanted background that is not related to PPIX fluorescence is slightly large in the other wavelength region of the first peak of PPIX fluorescence. In the FWHM of observation wavelength 1, small value of standard estimation error was obtained from about 10 to 50 nm. In these wavelength regions, spectral shapes of FAD and collagen were monotonically decreasing, while the PPIX fluorescence had almost no signal. In the FWHM of observation wavelength 3, small value of standard estimation error was obtained from 100 to 300 nm of FWHM. This result indicated that wide FWHM is better for the reliable estimation of the fluorescence intensity of PPIX due to feeble fluorescence intensity in the wavelength region longer than 700 nm. By performing the optimization, we finally obtained the optimum observation wavelengths of 536 nm with FWHM of 40 nm, 634 nm with FWHM of 20 nm, and 745 nm with FWHM of 204 nm. The minimum standard estimation error of PPIX fluorescence was 5.44.

For the actual experiment using a fluorescence microscope, we employed commercially available optical filters with near the optimum center wavelength and FWHM as follows: the center wavelength of 536 nm with FWHM of 40 nm (FF01-536/40–25; Semrock), 635 nm with FWHM of 18 nm (FF01-635/18–25; Semrock), and 736 nm with FWHM of 128 nm (FF01-736/128–25; Semrock). By using this filter set, the standard estimation error obtained was 5.85 under the same condition of the optimization as described above.

### Quantitative prediction of PPIX fluorescence intensity against adjacent chromophores

By using the optimized optical filter set, we demonstrated quantitative prediction of PPIX fluorescence intensity against autofluorescence. We prepared a concentration matrix of PPIX and FAD; the concentration of PPIX was varied from 0 to 100 nM with 25 nM step and that of FAD from 0 to 12 μM with 3 μM step.

**Figure 4:**
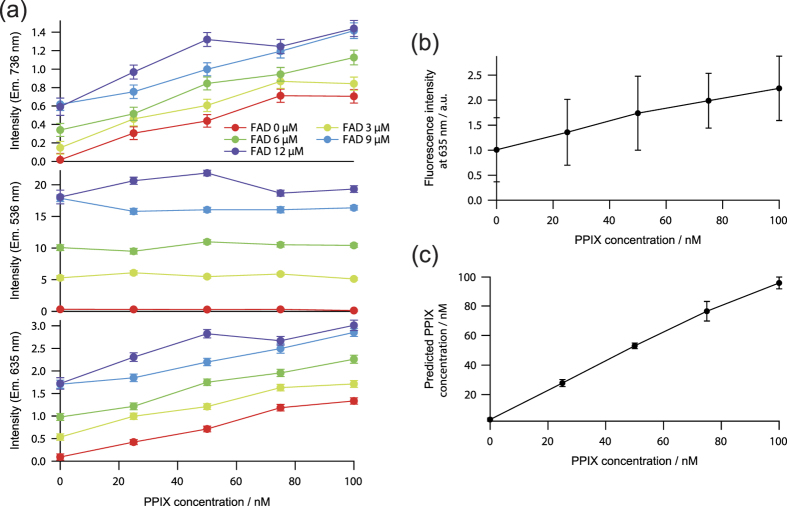
Background-free prediction of fluorescence intensities of PPIX in chemical mixtures. (**a**) Relation between fluorescence intensities observed at three observation wavelengths and the concentration of the chemical mixture. (**b**) Mean fluorescence intensity observed at 635 nm. (**c**) Predicted PPIX fluorescence by the calibration with three wavelength regions. Error bars represent standard deviation with the variation of the FAD concentration from 0 to 12 μM at each PPIX concentration.

The relation between fluorescence intensities observed at three observation wavelengths and the concentration of the complex mixture is shown in Fig. 4a. The concentration of PPIX did not affect to the fluorescence intensity at 532 nm. This result was obtained because the observation wavelength region of 536 nm with FWHM of 40 nm did not include the fluorescence spectrum of PPIX. In contrast, the fluorescence intensities at 635 and 736 nm linearly increased depending on the concentration of PPIX with the offset depending on the concentrations of FAD.

Mean fluorescence intensity observed at 635 nm, a conventional method for PPIX detection, and predicted fluorescence intensity of PPIX fluorescence calibrated by using three wavelength regions are shown in Fig. 4b,c. The regression coefficients of the three wavelength regions for the calibration of PPIX fluorescence were calculated by using the data shown in Fig. 4a. In the conventional PPIX detection method using only the wavelength of 635 nm, the variation of background autofluorescence of FAD affected the fluorescence intensity at 635 nm, resulting in prediction error of PPIX fluorescence. In the calibration method using the three observation wavelength regions, prediction error was greatly suppressed.

**Figure 5:**
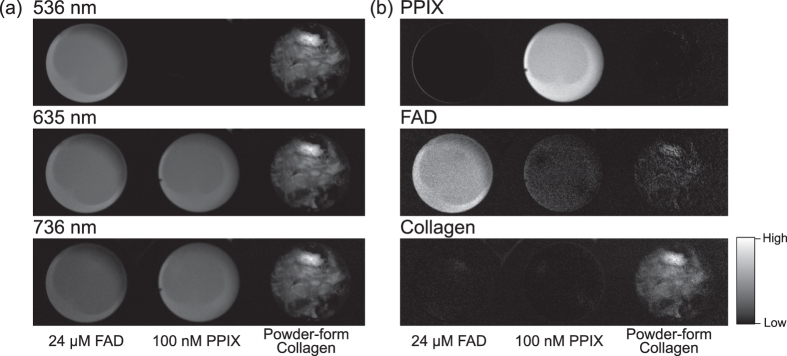
Selective imaging of PPIX fluorescence against other chromophores. (**a**) Fluorescence imaging of each wavelength region. (**b**) Selective imaging of PPIX against the other chromophores FAD and collagen by the calibration of three wavelength regions. Selective imaging of FAD and collagen in the same manner as the calibration is also shown.

We also demonstrated selective imaging of PPIX florescence by using 100 nM PPIX, 24 μM FAD, and powder-form collagen as shown in Fig. 5. In the conventional PPIX detection method using only the wavelength of 635 nm, fluorescence intensities of PPIX, FAD, and collagen were almost identical to each other, and thus the identification of PPIX was impossible (Fig. 5a). By calibrating the fluorescence intensities of three wavelength regions, PPIX fluorescence was markedly emphasized (Fig. 5b). Furthermore, by using the same three spectral images, our method also provided selective images of FAD and collagen as shown in Fig. 5b. These results indicated that our proposed method enabled background-free prediction of PPIX fluorescence and also the FAD and collagen fluorescence even in the presence of strong autofluorescence.

### Selective imaging of lymph node metastasis of colorectal cancer patients

**Figure 6:**
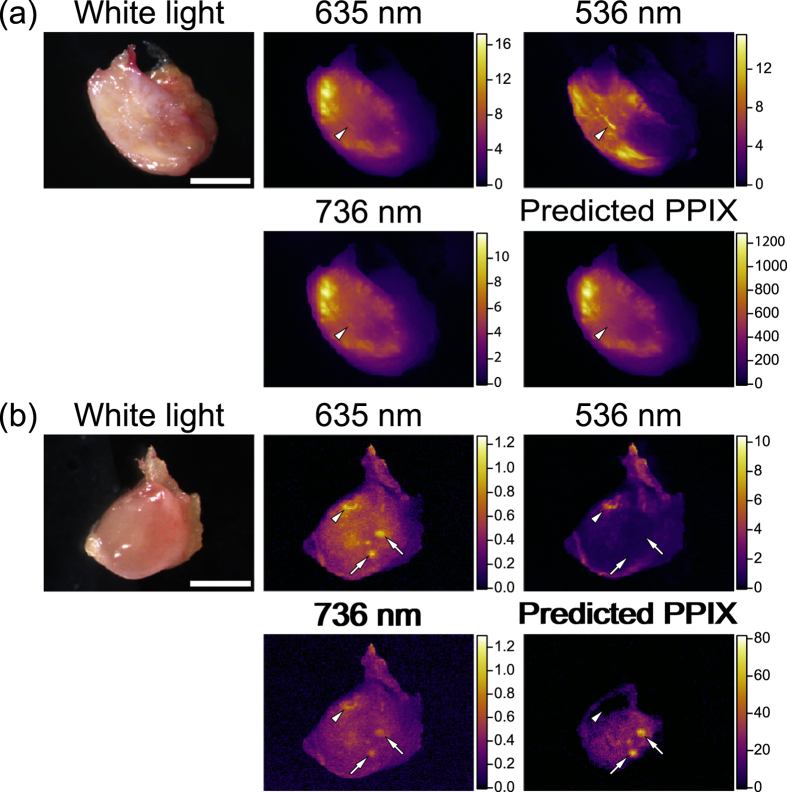
*Ex vivo* imaging of PPIX fluorescence of representative lymph node metastasis of a colorectal cancer patient. (**a**) Metastatic lymph node. (**b**) Non-metastatic lymph node. Arrowheads indicate unwanted autofluorescence other than PPIX, which was clearly eliminated in the calibrated images of PPIX fluorescence. Arrows show PPIX accumulation in non-metastatic regions, which may indicate follicles. Scale bars in a and b represent 5 mm and 2 mm, respectively.

We applied our method to metastatic and non-metastatic lymph nodes of human colorectal cancer patients. A total of 28 lymph nodes including one metastatic and 27 non-metastatic lymph nodes obtained from five patients were examined. Representative *ex vivo* images of lymph nodes are shown in Fig. 6. After lymphadenectomy in colorectal cancer surgery, we isolated lymph nodes and cut them in half. A cross-section of each lymph node was examined.

For the evaluation of lymph node metastasis, fluorescence intensity of PPIX and its spatial distribution are both important. In general, metastatic lesions exhibit strong fluorescence intensity and anisotropic distribution of PPIX. In fluorescence intensity observed at 635 nm, the metastatic lymph nodes exhibited stronger fluorescence than the non-metastatic lymph node. The spatial distribution of PPIX-predicted image of the metastatic lymph node was almost identical with that of the fluorescence image observed at 635 nm as shown in Fig. 6a because of the presence of PPIX that exhibited strong fluorescence at 635 nm in the metastatic lymph node. However, the small but notable difference of spatial distribution was detected as indicated by the arrow heads in Fig. 6a. Furthermore, in the non-metastatic lymph node, the spatial distributions of the fluorescence image observed at 635 nm and the PPIX-predicted image were clearly different, in which some confusing spatial distributions of the fluorescence intensity observed at 635 nm were obtained as indicated by the arrowheads in Fig. 6b. These anisotropic structures of fluorescence often misled us to infer the presence of metastatic lesions. Furthermore, the anisotropic structures also complicated the detailed evaluation of the distribution of malignant lesions even in the metastatic lymph node (Fig. 6a). In the calibrated image for the prediction of PPIX fluorescence by using three wavelength regions, these anisotropic structures were clearly eliminated, indicating that these regions might not be metastatic lesions, but autofluorescence such as from collagens and flavins. Round-shaped distribution of PPIX remained in the calibrated image as indicated by the arrows in Fig. 6b. These round-shaped distributions of PPIX were identified as possible lymphoid follicles. All these results supported the efficacy of our approach.

## Discussion

In this study, we proposed a noise-robust and simplified calibration method for background-free detection of PPIX fluorescence, especially for lymph node metastasis. We provided the optimum observation wavelength regions by computational simulation using representative fluorescence spectra of PPIX and typical background chromophores of FAD and collagen. We demonstrated the efficacy of background elimination using our method by using pure chemicals and metastatic lymph nodes excised from patients undergoing colorectal cancer surgery with lymphadenectomy. Once observation wavelength regions are optimized with the assumption of proper chromophores following our optimization method, the background-free detection of PPIX fluorescence can be realized with only a small number of spectral imaging. In the same manner, our method can be expanded to the other cases with the presence of other chromophores.

As mentioned, autofluorescence is a well-known noise source in PPIX fluorescence measurement, and several researchers have also proposed autofluorescence elimination methods, such as spectral peak detection^17^, spectral unmixing^18^,^19^, RGB color ratio imaging^20^,^21^, and photo-induced oxidization-assisted PPIX detection^22^. PPIX fluorescence has a strong unimodal peak at around 635 nm, while the other autofluorescence exhibits gently sloping spectrum. The spectral peak detection method identifies the PPIX spectral peak by observing fluorescence spectrum with a spectrometer or a hyperspectral imager. Spectral unmixing method is an improved version of the spectral peak detection method, and gives us quantitative fluorescence intensities of PPIX and autofluorescence by computationally separating multiple fluorescence spectra from merged fluorescence spectrum of PPIX and autofluorescence of tissues. These methods are, however, time-consuming for obtaining hyper-spectral images because three-dimensional images, two-dimensional space and one-dimensional spectrum are required. RGB color rationed imaging method is simple, and is easily realized by using a color CCD camera. Since PPIX fluorescence appears in the red region of the color CCD camera and the other autofluorescence mainly appears in the blue or green region, RGB color rationed images enable PPIX highlighting images. Although RGB color rationed imaging method realizes rapid imaging of PPIX fluorescence with only a single color CCD camera, quantitation of fluorescence intensity and sensitivity for malignant tumor is poor because non-optimized filters for PPIX detection are used in the commercially available color CCD camera. Photo-induced oxidization-assisted PPIX detection is based on unique photoconversion effect of PPIX to photoprotopropyrin (PPP) by blue light irradiation. There is a spectral peak of PPP at around 670 nm, while the peak at 635 nm in PPIX is decreased following blue light irradiation due to photobleaching. The intensity ratio of 670 nm to 635 nm thus is increased in PPIX following light irradiation, while that in other fluorescent chromophores such as collagen and flavins remains. This photoconversion method is effective for autofluorescence elimination, but it requires light irradiation for photoconversion. It takes about a few tens of seconds to minutes. Compared with the other methods, our proposed method has several advantages: the calibration of intensities in the optimal three wavelength regions sufficiently eliminates unwanted autofluorescence, small number of image acquisitions enables faster imaging, short total exposure is safe for the specimen, and *in vivo* imaging is available. Furthermore, our proposed method has potential for real-time imaging by employing a simultaneous observation method of three spectral images such as a 3-camera system, a simultaneous multi-spectral imaging system observing at the different regions of a single camera, or a customized color camera with the optimized color filters on the pixel array^24^.

Despite these advantages, however, some limitations remain. Firstly, our optimization is based on three typical fluorescence spectra, *i.e*., PPIX, FAD, and collagen. When the predominant contribution of autofluorescence is from FAD and collagen as in lymph nodes, our results are directly applicable and estimate the contribution of PPIX, FAD and collagen as shown in Fig. 6. In the other tissues with the other chromophores, our approach can also be applicable after the recalculation of the optimum observation wavelength following our scheme described above. If the contribution of the autofluorescence of the chromophores other than FAD and collagen is sufficiently low or can be spectrally fitted with the spectra of FAD and collagen used in this study in the wavelength regions we optimized, quantitative detection of PPIX fluorescence can directly be performed with our results. Further studies, however, should examine a large number of specimens excised from patients to confirm the reliability of our method in various tissues types. Moreover, blood is a strongly absorbing material for both the excitation and emission light for PPIX estimation. If blood is present around the malignant tumors examined, estimation accuracy of PPIX fluorescence may decrease. To eliminate the effect of blood on fluorescence observation, careful and complete washing out of blood in fluorescence observation or spectral analytical methods for the elimination of this effect is required. Thirdly, several dyes, such as indocyanine green, indigo carmine, ink, and so on, are sometimes used for the identification of malignant tumors or tissue species. These dyes may also affect fluorescence observation of PPIX similarly to blood. The effect of these dyes on the detection accuracy of PPIX fluorescence is still unclear, and thus should be examined in further study when our proposed method is applied to cancer surgery with the use of dyes. Finally, our method requires three individual images at least. For the accurate estimation of PPIX fluorescence with these images, one is required to maintain the same observed region among these images. When a movable specimen is observed such as in *in vivo* imaging, additional methods to maintain the same observed area are required, such as mechanical fixation of the specimen, an automatic tracking algorithm of the relative position of the images, and simultaneous observation of images with multiple cameras.

## Conclusion

This study examined simplified multispectral imaging for quantitative detection of PPIX fluorescence. Elimination of autofluorescent background is essential for accurate evaluation of malignant tumor by using 5-ALA-based fluorescence diagnosis. We envisage that integration of this technique with a surgical microscope or further development of *in vivo* imaging system for intraoperative use will yield rapid, accurate, and intraoperative diagnosis of malignant tumors for minimally invasive surgery in the future.

## Methods

### Computational optimization of observation wavelength

We utilized matlab software (Mathworks, Natick, MA, USA) for computational simulation to seek optimum wavelength of PPIX fluorescence detection. The representative fluorescence spectra of PPIX (P8293; Sigma-Aldrich, St. Louis, MO, USA), FAD (194664; Wako Pure Chemical Industries, Osaka, Japan), and collagen type-I (C9879; Sigma-Aldrich, St. Louis, MO, USA) used for the optimization of observed wavelength were obtained by an intensified multichannel spectrometer (MCPD-7000; Otsuka Electronics, Osaka, Japan) mounted on a stereoscopic microscope (SZX12; Olympus, Tokyo, Japan) with sufficient signal-to-noise ratio by excitation with a mercury lamp (U-LH100HG; Olympus, Tokyo, Japan) via 405 nm optical filter (BP400-410; Thorlab, Newton, NJ, USA).

### Fluorescence imaging

A stereoscopic fluorescence microscope (SZX16; Olympus, Tokyo, Japan) was used for acquiring fluorescence. A mercury lamp (USH1030; Olympus, Tokyo, Japan) was employed as light source for fluorescence excitation. The wavelength of excitation light was set at 405 nm by using an optical filter (BP400-410; Thorlab, Newton, NJ, USA), and the excitation light illuminated with the energy density of 1 mW/cm^2^ in all experiments. Fluorescence from the sample was corrected with an objective lens (×0.5; Olympus, Tokyo, Japan). The total observation magnification was set at ranging from ×0.35 to ×5.75. The fluorescence was obtained with a monochrome charge coupled device (CCD) camera (ORCA-ER; Hamamatsu Photonics, Hamamatsu, Japan) with the exposure time of 1 s via the optimized fluorescence filters with the center wavelength of 536 nm (FF01-536/40–25; Semrock, IDEX, Lake Forest, IL, USA), 635 nm (FF01-635/18–25; Semrock, IDEX, Lake Forest, IL, USA), and 736 nm (FF01-736/128–25; Semrock, IDEX, Lake Forest, IL, USA) as described in this article.

### Pure chemical experiment

For concentration dependency observation, we freshly prepared stock solutions of 0.1 μM PPIX dissolved in dimethyl sulfoxide (DMSO, C9879; Sigma-Aldrich, St. Louis, MO, USA) and 24 μM FAD dissolved in DMSO. The DMSO was used as solvent of PPIX and FAD to make well-dissolved solutions of several concentrations. The PPIX stock solution, the FAD stock solution, and DMSO were mixed depending on observed concentrations in microtubes. A total of 120 μL of the mixed solution was poured at each well of a 384-well plate (242764; Nunc, Thermo Fisher Scientific, Waltham, MA, USA).

For the comparison of fluorescence of PPIX, FAD, and collagen, 0.1 μM PPIX dissolved in DMSO, 24 μM FAD dissolved in DMSO, and collagen powder from bovine Achilles’ tendon (Sigma-Aldrich, St. Louis, MO, USA) were placed on each well of a 96-well plate (12-566-72; Nunc, Thermo Fisher Scientific, Waltham, MA, USA).

### Human lymph node experiment

All the aspects of the study were approved by the Ethics Committee of Kyoto Prefectural University of Medicine (Permit Number: RBMR-C-671-1), and all patients provided written informed consent. The study protocol conformed to the ethical guidelines of the Declaration of Helsinki.

Lymph nodes obtained from patients undergoing colorectomy with lymphadenectomy at University Hospital, Kyoto Prefectural University of Medicine were examined. 5-ALA hydrochloride (AL-05-1; Cosmo Bio, Tokyo, Japan) at 15 mg/kg of body weight dissolved in 20 mL of 50% glucose was given to patients by oral administration at 2 h prior to surgery. The patients were protected from direct sunlight for 24 h after administration of 5-ALA. Lymph nodes were operatively resected en bloc with the primary tumor, and isolated from adjacent tissues. The resected lymph nodes were protected from light until fluorescence observation. The lymph nodes were cut in half just before the fluorescence observation. Resection of lymph nodes and fluorescence observation were completed in 5–9 hours after 5-ALA administration. After the fluorescence observation, we confirmed the histology of the lymph nodes with routine hematoxylin and eosin staining by the pathologists who were the co-author of this article.

## Additional Information

**How to cite this article**: Minamikawa, T. *et al.* Simplified and optimized multispectral imaging for 5-ALA-based fluorescence diagnosis of malignant lesions. *Sci. Rep.* **6**, 25530; doi: 10.1038/srep25530 (2016).
